# Experiential interprofessional education for medical students at a regional medical campus

**Published:** 2018-03-27

**Authors:** Laura Walmsley, Melanie Fortune, Allison Brown

**Affiliations:** 1 Michael G. DeGroote School of Medicine, Niagara Regional Campus, McMaster University, Ontario, Canada; 2 Department of Health Research Methods, Evidence, and Impact, McMaster University, Ontario, Canada

## Abstract

**Background:**

Regional medical campuses are often challenged with providing effective interprofessional education (IPE) opportunities for medical students that are comparable to those at main campuses. At distributed teaching sites, there is often less IPE infrastructure and fewer learners of other health professions. On the other hand, distributed medical education (DME) settings often have community-based clinical environments and fewer medical students, which can provide unique opportunities for IPE curriculum innovation.

**Methods:**

At the Niagara Regional Campus (NRC) of McMaster University, the Horizontal Elective for Interprofessional Growth & Healthcare Team ENhancement (HEIGHTEN) was developed to provide first-year medical students the opportunity to learn from and work alongside nurses in a community hospital. This study assesses HEIGHTEN’s impact on students’ knowledge, confidence, and attitudes towards interprofessional care, as well as student satisfaction with the learning experience using a mixed methods evaluation.

**Results:**

Findings suggest that HEIGHTEN provided an enjoyable learning experience, fostered positive interprofessional attitudes and an appreciation for the nursing role. Voluntary participation by medical students was high and increased both within the regional campus and with students from other campuses travelling to participate.

**Conclusion:**

This model for IPE can be feasibly replicated by distributed teaching sites to provide medical students with hands-on, experiential learning early in training, leading to positive attitudes and behaviours supporting interprofessional collaboration (IPC).

## Introduction

Interprofessional collaboration (IPC) is considered by researchers, healthcare organizations, educators, and governments as integral in the provision of safe, effective, and efficient healthcare.^1-5^ Effective interprofessional education (IPE) can foster future IPC by facilitating knowledge of others’ roles, respectful and positive attitudes towards other professionals, and interdisciplinary communication skills.^6-9^ Despite evidence surrounding the importance of IPE and IPC, educational institutions often struggle to develop and implement effective interprofessional training strategies.^10^

The literature suggests that authentic, clinical experiences are critical to fostering student understanding of and engagement in IPE and IPC.^8,9^ This is preferable to passive lecture style interprofessional training, as it allows students to connect formal knowledge to clinical experience and encourages practical learning and practice change.^11^ A review of IPE in Canadian medical schools by the Canadian Federation of Medical Students (CFMS) in 2013 found that while all schools offer IPE learning opportunities that meet accreditation standards, few medical schools make optimal use of interactive learning opportunities in clinical settings within their IPE curriculum.^12^

Through working alongside mostly or only physicians, medical students often gain exposure to clinical settings early in their training. However, experiential IPE with non-physician healthcare professionals is often not integrated into their training until clerkship.^13-15^ Evidence supports that most students are able to identify the roles of their own profession relative to others early on in clinical settings,^16^ so this is no reason to delay IPC exposure until later training. Research also affirms that student attitudes and perceptions about other healthcare professions can be shaped by IPE.^8,9^ This is especially critical during early training.

### McMaster University’s IPE curriculum

At McMaster University, situated in Hamilton, Ontario, Canada, each medical student completes a mandatory IPE curriculum. There are three levels of IPE experiences from which medical students must obtain credits: exposure, immersion, and mastery. Exposure credits are shorter experiences that aim to introduce medical students to healthcare roles, and are available through special events such as “lunch and learns” on healthcare topics. Immersion credits are longer in duration, and usually involve interaction between students of different professions. For example, McMaster hosts an “IPE Day” where all health professional students attend a conference in Hamilton to discuss IPC and explore the scope and practice of other professions. Students are also required to shadow a non-physician healthcare provider in a home visit to a patient as part of the immersion level. Mastery credits are only available during clerkship, and require that students document engagement in IPC by completing six “interprofessional encounter cards” with non-physician health professionals.

A rudimentary needs assessment conducted in March 2015 of McMaster University pre-clerkship medical students (N=33) found that despite an emphasis placed on quality IPE and healthcare collaboration within McMaster’s Faculty of Health Sciences, students perceived a gap in their knowledge of IPE and their ability to effectively collaborate within healthcare teams. Of the students who responded to the survey, 82% (N=33) expressed a desire to know more about other healthcare professionals and how to best collaborate with them. Additionally, 76% (N=33) felt that opportunities to work alongside healthcare professionals during their first year of training would be helpful in addressing this gap. These findings were used to guide the development of an IPE intervention using quality improvement principles emulated from the Model for Improvement as part of an extracurricular offering at McMaster University.^17,18^

### IPE challenges at the Niagara Regional campus

The Niagara Regional Campus (NRC) of the Michael G. DeGroote School of Medicine at McMaster University is located in St. Catharines, Ontario, approximately one hour from the main teaching campus in Hamilton, Ontario. Medical students who end up at NRC begin their three years of medical training with three months at the main campus in Hamilton. The remainder of the pre-clerkship curriculum and core clerkship rotations are then completed at NRC, meaning that students spend approximately 30 months in the Niagara region to complete their classroom and clinical experiences.

The main campus in Hamilton is home to many health professions programs, including medicine, nursing, midwifery, social work, occupational therapy, physical therapy, and physician assistants. Students from these professions complete their classroom components on campus and the majority of clinical rotations in the Hamilton area. IPE events that occur throughout the year on the main Hamilton campus bring together students from these programs. In contrast, Niagara is home to fewer health professions programs – nursing students from Brock University and Niagara College, and Rehab Assistant students (Occupational Therapy & Physiotherapy Assistants) from Niagara College. As a result, classrooms and clinical teaching sites in Niagara have fewer learners, and learners from fewer professions. Overall, regional campus students not only have less opportunity to engage with the main IPE curriculum at McMaster’s main campus, but are also exposed to fewer non-physician learners even during their clinical years. These shortcomings may be common amongst distributed medical education (DME) environments and propel the creation of innovative means to teach IPE effectively in these settings.

### The Horizontal Elective for Interprofessional Growth & Healthcare Team ENhancement (HEIGHTEN)

The Horizontal Elective for Interprofessional Growth & Healthcare Team ENhancement (HEIGHTEN) is a student-developed and student-led program that was created to meet the needs of first-year medical students at NRC who experienced challenges in accessing high-quality, meaningful IPE. HEIGHTEN seeks to enhance McMaster’s current pre-clerkship IPE curriculum by creating early experiential IPE training opportunities where first-year medical students learn from and work alongside non-physician healthcare professionals.

During HEIGHTEN, each medical student works with nurses for four to eight hours on a general medicine floor of a community hospital in St. Catharines, Ontario. A strong emphasis is placed on the interactive and hands-on nature of the program, promoting a more engaging experience than an observership or shadowing opportunity. Each student participates in the daily clinical duties of nursing staff including IPE rounds, providing personal care, transferring patients, placing IVs, and administering medications. Students perform all tasks with supervision and direct observation or assistance from their nursing preceptor. Nurses are instructed to use their skills for teaching nursing students, which they have acquired through their formal education and clinical experience, to actively engage medical students in their day-to-day tasks as they would a nursing student. Brief training sessions are provided to nursing staff by their clinical manager to explain the stage of training of first-year medical students. A one-page handout summary of HEIGHTEN is also distributed amongst nursing staff and provided by the medical students at the beginning of their shifts.

The purpose of this study was to explore the impact of experiential, pre-clerkship IPE programming involving medical students working with nurses in the provision of health services in a DME setting on students’ knowledge, confidence, and attitudes towards interprofessional care, as well as student satisfaction with the learning experience.

## Methods

There was a 71% participation rate in the inaugural offering of HEIGHTEN from January to June 2016, with 20 of the 28 first-year students in the Class of 2018 from NRC participating. These students had not participated in any previous experiential IPE activities and volunteered to participate in HEIGHTEN without academic IPE credit or incentive. To understand the impact of HEIGHTEN on pre-clerkship medical students, a mixed-methods approach was used to determine medical students’ knowledge, skills, and attitudes towards IPE and IPC. Each participant was asked to complete a pre- and post-HEIGHTEN survey, which included five questions assessing their confidence with IPC. These five questions were emulated from several existing instruments that have been previously used in the IPE literature: the Team Skills Scale,^19^ the Attitudes Towards Health Care Teams (ATHCT) scale,^20^ and the Self-Efficacy for Interprofessional Experiential Learning (SEIEL) tool.^21^ Some questions from each existing scale were compiled and modified to reflect the learning opportunities created by HEIGHTEN. Each of the five items included the same 10-point adjectival response option to assess degree of confidence, ranging from “Not Confident Whatsoever (1)” to “Totally Confident (10).” Raw data were entered into SPSS 23 (IBM Corp, 2014) and analyzed using a paired *t*-test to detect statistically significant differences following HEIGHTEN. Since only two means for each item (pre- and post-HEIGHTEN) are compared, an Analysis of Variance (ANOVA) test with a post-hoc *t*-test was not performed, as the study sample is too small to conduct an ANOVA. Instead, a Bonferroni correction was used to compensate for the increase in Type I errors given the multiple hypotheses tested in the paired samples t-test. Cohen’s D statistic was calculated to examine the effect size for each item. Cronbach’s alpha was calculated to assess psychometric properties of internal consistency for the 5-item instrument.^22^

To assess student satisfaction, students were also asked whether or not they would recommend HEIGHTEN to another student in the post-HEIGHTEN survey. Open-ended survey questions were also asked to determine student satisfaction with the program as well to understand their attitudes and behaviours towards IPC following HEIGHTEN. These locally-developed, open-ended questions were included to allow participants to further comment on their experience in their own words, and were analyzed using content analysis.

In addition, two focus groups were held in May 2016 with HEIGHTEN participants. The purpose of the focus groups was to gain insight into the student experience from the perspective of participants that was in addition to the qualitative data obtained from the surveys, as well as to obtain program evaluation information by elucidating the benefits and limitations of this program as experienced by first-year medical students. These focus groups were conducted by an investigator at arm’s length from HEIGHTEN, who has graduate-level training in qualitative methods. The first focus group contained 10 participants, while the second focus group contained a smaller group of five students, representing 75% of HEIGHTEN participants in this aspect of the study. Both focus groups were held in-person on the medical school campus, and were audio-recorded and transcribed. Qualitative data from the focus groups were analyzed using thematic analysis by an investigator who was at arm’s length from the program.^23^ This initially involved close reading of the transcripts to familiarize the researcher with the data, allowing initial themes to emerge inductively. Then, these themes served as a coding framework which were then applied to the transcripts as they were re-read, and themes were further refined and applied to the data. The refined themes were then extracted to analysis as “nodes” in Nvivo 10 (QSR International, 2012) for final analysis and interpretation.

## Results

**Figure 1:**
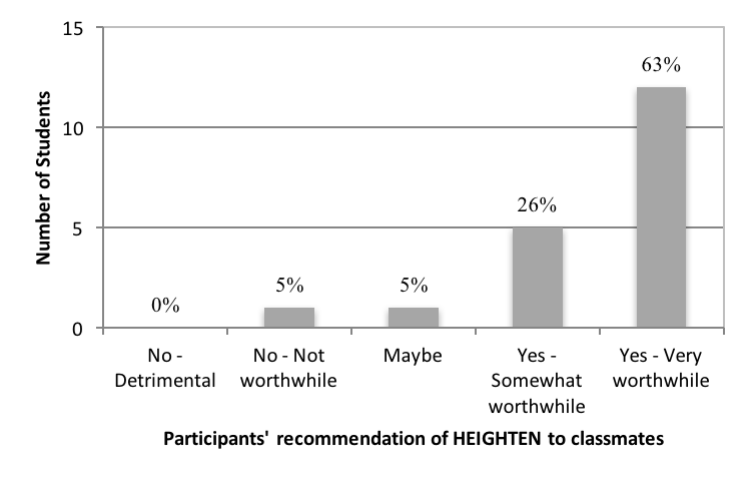
Student satisfaction with HEIGHTEN

Nineteen medical students who participated in HEIGHTEN completed the survey instruments, representing a 95% response rate. Overall, this experiential, clinical IPE opportunity was well received by participants. Of the students who participated in the first cycle of HEIGHTEN, 90% stated that they would recommend the elective to a classmate (Figure 1).

Confidence in IPC improved significantly following HEIGHTEN across all five questions (p<.01) (Table 1), which were compiled and modified from existing scales as described above. The largest mean increase and effect size was seen in student confidence in their understanding of when and how to collaborate with nurses as a medical student to improve patient care. Large effect sizes (d>0.8) resulted for each of the five instrument items. Cronbach’s alpha was 0.844 for the combined pre- and post-HEIGHTEN scores. When calculated separately, the pre-HEIGHTEN Cronbach’s alpha was 0.759, and post-HEIGHTEN alpha was 0.846, suggesting that the five questions assessing confidence towards IPC has high internal consistency among subjects.

**Table 1:** Paired t-test of the 5-item questions assessing confidence towards IPC

	PRE	POST	Δ	95% Confidence Interval			Effect Size**
	
	Mean (SD)	Mean (SD)	Mean (SD)	Lower	Upper	p-value*	D (upper, lower)
1. Your current knowledge of the roles and scope of practice of the nursing profession	5.05 (1.58)	7.47 (1.26)	2.42 (1.39)	1.75	3.09	<.001*	1.74 (1.17, 2.45)
2. Your ability to talk to a nurse as a medical learner (about things you don’t know, aspects of patient care, etc.)	6.79 (1.81)	8.52 (1.35)	1.74 (1.91)	0.816	2.66	<.001*	1.11 (0.51, 1.93)
3. Your understanding of when and how to collaborate with the nursing profession as a medical learner to improve patient care (e.g., consultations, asking other opinions or assessments of patient’s status)	5.00 (1.41)	7.95 (1.18)	2.95 (1.39)	2.28	3.62	<.001*	2.33 (1.80, 2.97)
4. Your ability to learn from and work together with non-physician professions (e.g., OT, PT, pharmacist, social worker)	5.68 (1.60)	7.74 (1.24)	2.05 (1.62)	1.27	2.83	<.001*	1.48 (0.92, 2.20)
5. Your ability to identify contributions to patient care that different disciplines offer	5.37 (1.83)	7.74 (1.24)	2.37 (1.98)	1.42	3.32	<.001*	1.56 (1.00, 2.38)

Adjectival scaling responses; 1 = Not confident at all, 10 = Totally confident

* two-tailed test of significance using a Bonferroni correction, α = 0.01

** Cohen’s D effect size

Qualitative data from the open-ended questions on the HEIGHTEN survey, as well as the two focus groups shed light on the impact of HEIGHTEN on medical students. Findings from students’ qualitative post-elective survey data first revealed that students shared appreciative attitudes towards the nursing role. When asked what they had learned from their experience, one student wrote:

How critical nurses are to efficient patient care, the difference that a caring nurse can make in the lives of their patients. [I] was surprised by how much nurses knew about physiology and pathophysiology.

Further, an emphasis on the non-physician dyad contributions to patient care experiences was evident. For instance, one student commented that the part they enjoyed most about HEIGHTEN was *“seeing the day-to-day lives of the nurses and patients and learning where physicians fit into the picture.”* To identify the nurse-patient dyad as experiencing a day-to-day life experience, with the physician contribution being secondary, indicates impact on the perspective of medical learners who may otherwise only appreciate the physician role as a central contributor to patient care.

Lastly, students’ intention to improve collaboration with interprofessional teams in future practice was the final theme amongst the survey data. One shared:

It was very hands-on and it also gave some insight into nursing scope of practice, and highlighted a couple of ways in which health care teams (especially physicians) can optimize orders or minimize inconvenience for nurses.

Findings from analysis of the focus group transcripts were congruent with our findings from the open-ended survey data. The experiential aspect of HEIGHTEN was successful in engaging medical students in day-to-day nursing activities, allowing them to see patient care from the perspective of a nurse. One student described a profound experience while helping a nurse bathe and feed a patient, when they realized that patient care involved more than providing medical treatment, stating:

I wasn’t there to provide medical care in the traditional sense - in the way we’re being trained right now. I was there to help the patient feel truly cared for, and this went beyond my understanding of patient care.

This hands-on experience learning about the nursing role from nurses not only allowed medical students to better understand and appreciate the role of nursing, but also motivated them to become better collaborators in the future. Several students commented that they learned what *not* to do in the future from this experience by having discussions with the nurse about their role, including the importance of clear, timely communication with the interprofessional team. Specifically, following HEIGHTEN participation, students felt more confident in approaching nurses for help or with questions, but also noted the importance of boundaries, respecting the nursing scope and busy schedules. All students agreed that this experience will influence how they interact with nurses in the future.

Following the first cycle of HEIGHTEN, the program became a recognized McMaster medical school IPE credit and now provides added academic incentive to students who participate in future iterations. During the second cycle in 2017, an increased participation rate of 86% (N=28) was achieved at NRC with 24 participants. Additionally, 27 students from the main campus in Hamilton and one student from the other regional campus in Waterloo travelled to Niagara to participate, doubling the participation level from the first iteration. As of June 1 2017, 52 students in the Class of 2019 have voluntarily completed HEIGHTEN, which is over 25% (N=203) of the student body.

## Discussion

Effective IPE is a prerequisite for collaborative future healthcare teams,^6-9^ and is therefore an integral component of high quality medical education. HEIGHTEN provides a hands-on interprofessional learning opportunity for first-year medical students that is feasible in DMEs. During HEIGHTEN’s first year of implementation, there was high voluntary participation by regional campus students despite the initial lack of academic incentive for participation. Participation subsequently increased in its second year, demonstrating continued strong student engagement in this IPE offering. Furthermore, high student satisfaction ratings related to the IPE learning experience that HEIGHTEN provided suggest that it is an appreciated and highly sought-after program within the medical school curriculum. The large effect sizes observed in this study suggest that student confidence across multiple IPE domains was significantly enhanced by their participation in HEIGHTEN. The success of HEIGHTEN suggests that early, experiential IPE can be effective. This is consistent with the literature, which tends to emphasize hands-on learning as preferable to passive lecture style teaching for IPE in medical education.^8,9,11,12,15^

Most IPE programs reported in the literature include passive lecture style teaching methods during pre-clerkship, and focus on student-to-student interactions from a range of health professions.^10^ That being said, two published American studies from the University of Vermont^24^ and the University of Michigan^25^ were found, which outline innovative approaches to IPE similar to HEIGHTEN, whereby first-year medical students shadowed nurses. Both of these studies demonstrated positive interprofessional outcomes including enhanced knowledge of the nursing profession, an understanding of the importance of interprofessional communication, and improved medical student attitudes toward nurses. Several Canadian medical institutions have also implemented shadowing opportunities for medical students, whereby they observe the role of another healthcare professional in a clinical setting. For instance, the University of Toronto piloted a program where third-year medical students were assigned to shadow a non-physician healthcare professional for a two-hour period.^26^ Similarly, McMaster University has a mandatory home visit program prior to clerkship, where students shadow a nurse or allied health professional in a community setting. On the other hand, early and experiential IPE training where medical students work with non-physician professionals in a hands-on (not merely “shadowing”) manner is uncommon at many Canadian medical schools.

### Experiential IPE in Distributed Medical Education (DME)

HEIGHTEN presents a novel opportunity for supporting high quality experiential IPE in the context of DME. McMaster’s regional medical campuses have unique barriers as well as opportunities for IPE. Many IPE school events require regional campus students to travel or use video conferencing to participate. There are fewer non-medicine learners at the hospitals affiliated with regional campuses, which limits student-to-student IPE. Though, this also means there are low learner-to-clinical staff ratios, which made HEIGHTEN a more effective and enjoyable learning experience. Further, HEIGHTEN requires minimal extraneous funding and is straightforward from an administrative standpoint relative to other IPE programming, enhancing feasibility in the DME setting.

Unlike most IPE training that often primarily targets interactions between students of different professions, HEIGHTEN places students with professionals in their workplace. Although there is some evidence of positive learning outcomes from interdisciplinary student interaction, learner behaviour change is infrequently seen.^14^ IPE that provides opportunities for lived experiences in other professional roles offer superior learning experiences and are more useful to students as they move forward in their careers.^12,14^ Lastly, HEIGHTEN provides added benefits to participants that are outside the realm of IPE and IPC. The hands-on nature of HEIGHTEN has led to student participation for the dual purpose of enhancing their IPC skills *as well as* acquiring valuable clinical skills, patient interaction experience, and orientation to inpatient clinical settings that will help to prepare them for clerkship. This is an important aspect of HEIGHTEN that has motivated strong student engagement in this IPE offering.

### Limitations

This study is limited by the small sample size of participants, and lack of control or comparison groups which limit statistical inferences and generalizations. However, the large effect sizes observed in our data suggest a positive influence of HEIGHTEN on medical student confidence to engage in interprofessional collaboration. Additionally, the convenience sample of participants includes medical students who willingly participated in HEIGHTEN outside of the traditional IPE curriculum, and thus may be inherently more interested in IPE or susceptible to changes in their knowledge, skills, and attitudes towards IPC. The qualitative aspect of this study was conducted with one researcher, meaning that the analysis did not involve any aspect of triangulation from another investigator. However, the results that emerged from the qualitative analysis were congruent with the findings from the quantitative analysis. Furthermore, examining how HEIGHTEN may influence medical students’ ability to collaborate and demonstrate IPC competencies in an applied manner has not yet been evaluated, but could be assessed in future research using the Team Observed Structured Clinical Encounter (TOSCE).^27^ Future research will study the impact of HEIGHTEN in comparison to traditional IPE credits at McMaster University (e.g., IPE day), and on later abilities to collaborate in a team-based, interprofessional setting during the clinical years of medical school.

### Conclusions

Regional medical campuses face unique challenges in the delivery of effective IPE. At the same time, DME sites can be conducive environments for curriculum innovation. Clinical placements with nurses is an effective approach to promote meaningful IPE at an early stage of training. HEIGHTEN is a program offering which can be emulated by other Canadian medical schools, especially at distributed campuses to provide local, hands-on IPE learning opportunities. This program can promote the integration of experiential, relevant IPE in the pre-clerkship curriculum to cultivate early appreciation for the nursing role, positive behaviours towards IPE and IPC, and collaborative interprofessional relationships within healthcare teams.
